# The Effects of a Phytochemical Supplement Blend on Markers of Exercise-Induced Muscle Damage: A Randomised Controlled Trial

**DOI:** 10.3390/nu18081199

**Published:** 2026-04-10

**Authors:** Josh Thorley, Kirsty M. Reynolds, Matt Nickels, Stephen J. Bailey, Ronald Kingma, Tom Clifford

**Affiliations:** 1 School of Sport, Exercise and Health Sciences, Loughborough University, Loughborough LE11 3TU, UK; 2 School of Translational Medical Sciences, University of Nottingham, Nottingham NG7 2QL, UK; 3 VDF FutureCeuticals Inc., Momence, IL 60954, USA

**Keywords:** polyphenol, exercise recovery, antioxidant

## Abstract

Background and Objectives: This study examined the effects of a novel phytochemical supplement blend on markers of exercise-induced muscle damage. Methods: In a randomised, parallel group design, 24 healthy participants (14 males) consumed 300 mg of a phytochemical blend (calcium fructoborate, turmeric and pomegranate; PB) or inert placebo for 9 days (n = 12 per condition). On day 7, participants performed 150 drop jumps to induce muscle damage. Markers of neuromuscular function, muscle soreness/pain, perceived exhaustion and sleep quality, were measured pre-exercise and 24, 48, and 72 h post-exercise; systemic markers of inflammation, muscle damage, and oxidative stress were measured on these days as well post-exercise and 2.5 h post-exercise. Results: There was an interaction effect for pressure pain threshold in the vastus lateralis (*p* = 0.041), which was ~21% higher in PB 72 h post-exercise (*p* = 0.074; *d_s_* = 0.767). Perceived sleep quality was greater 72 h post-exercise in PB (*p* = 0.049; *r*_rb_ = 0.423) and those in the PB condition reported feeling more recovered and less mentally drained post-exercise (*p* ≤ 0.043). There were no statistically significant between-condition differences for any markers of neuromuscular function, inflammation, oxidative stress or muscle damage (*p* > 0.05). Conclusion: In conclusion, a novel PB showed promise for attenuating muscle pain and perceived exhaustion, and improving sleep quality, in the days after muscle damaging exercise. The study protocol was pre-registered on the Open Science Framework Registry (registration number: qgw3a).

## 1. Introduction

Strenuous exercise that is unaccustomed or involves forceful eccentric muscle contractions can damage skeletal muscle [1,2]. The initial injury evokes an acute inflammatory response that, in repairing and remodelling the damaged structures, may cause further damage to muscle proteins in a process termed ‘bystander injury’ [3,4]. Outwardly, this manifests as decreases in neuromuscular performance and increases in muscle soreness, which can last for several days depending on the severity of the muscle damage [4,5]. The associated muscle soreness, reduced range of movement, and more painful movement may also negatively impact sleep quality. This, in turn, has been shown to exacerbate muscle soreness [6,7], possibly further delaying functional recovery and negatively affecting exercise training quality and competition in the ensuing days. Consequently, there is great interest in strategies that can attenuate these symptoms and accelerate the return to pre-exercise function.

A popular strategy to expedite recovery following muscle-damaging exercise is to provide bioactive dietary interventions with anti-inflammatory and antioxidant effects. Several phytochemicals, most notably (poly)phenols, have been shown to possess these biological effects [8]. Various mechanisms have been proposed to explain how these compounds exert these effects, including activation of nuclear factor-erythroid-2-related factor 2 (NRF2), which upregulates redox enzymes [9,10], decreasing oxidative stress [11] and disrupting of pro-inflammatory signalling pathways, which restricts cytokine secretion and the inflammatory response [8,12]. While the data are equivocal, dietary interventions containing anthocyanins [13], quercetin [14], ellagitannins [15] and curcumin [16] have all shown some promise in human clinical trials of attenuating markers of exercise-induced muscle damage (EIMD). Additionally, calcium fructoborate, a highly characterised and nature-identical plant mineral complex known primarily for its potential benefits related to joint health [17,18], has been reported to possess antioxidant and anti-inflammatory effects [19].

Despite the initial promising results with individual phytochemicals or foods, there is a dearth of studies examining the effects of multi-ingredient supplements on markers of EIMD. In addition, most studies have been designed to provide relatively high doses requiring the intake of several capsules at once, or multiple doses per day [20,21], which may not be realistic or practical for consumers. Several reports have indicated that calcium fructoborate can reduce pain and inflammation as well as improve mobility in non-exercising populations at doses from 108 to 216 mg/day [19]. Therefore, providing calcium fructoborate in combination with selected botanical extracts containing phytochemicals with antioxidant and anti-inflammatory effects could be more effective than providing any single compound or phenolic compounds extracted/concentrated from one botanical source. Furthermore, while some studies provide phytochemical supplements in beverage format instead of capsules, a limitation of most drinks is that due to their unique flavour (e.g., cherry juice, beetroot juice), they cannot be easily taste matched for study blinding. In addition to the challenges of flavour matching, active and control beverages require detailed alignment of all macro and micronutrients as well as phytochemicals.

Therefore, the aim of this study was to examine the effects of a novel blend of calcium fructoborate and selected fruit extracts rich in phenolic phytochemicals, in a relatively low-dose encapsulated supplement format, on markers of EIMD. We hypothesised that the intervention would attenuate markers of EIMD (neuromuscular performance, muscle soreness, inflammation, oxidative stress) as compared to a placebo control. Secondarily, we hypothesised that the intervention would result in improved sleep quality and reductions in exercise-induced fatigue compared to the control.

## 2. Materials and Methods

### 2.1. Participants

**Figure 1:**
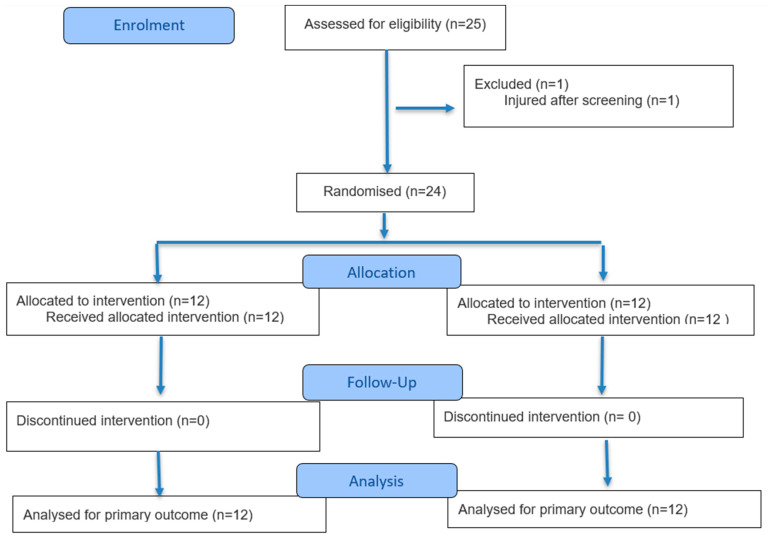
CONSORT flow diagram of study procedures.

Twenty-four healthy males (*n* = 14) and females (*n* = 10), who were classed as Tier 1 (‘recreationally active’) in the participant classification framework [22] volunteered for this trial. All participants met the World Health Organisation’s minimum activity guidelines and participated in multiple forms of activity, including ≥2 strength training sessions per week. The physical characteristics of the participants in the two conditions are presented in Table 1. See Figure 1 for the CONSORT flow diagram for the enrolment, allocation, and follow up details. All participants provided written informed consent and completed a health screening questionnaire prior to study entry. Participants were excluded if they had a known food allergy, had currently, or recently used anti-inflammatory medications (within 1 month of participation), were suffering from a musculoskeletal injury, or any disease or condition that was contraindicated by the study procedures. Participants were instructed to avoid consuming any dietary supplements or using putative recovery aids (e.g., compression garments) throughout the trial. Exercise outside of the trial was restricted from 36 h before the exercise bout to the end of the data collection. As in previous studies [23,24], female participants’ menstrual cycle phase was estimated subjectively with a survey. Four female participants were normally menstruating; the remaining six participants used monophasic oral contraceptives (n = 4), a copper iud (n = 1), or an implant (n = 1). All testing was scheduled to take place during the follicular phase (day 1–10 after first bleed) of their menstrual cycle or where applicable during the first 10 days of pill withdrawal.

### 2.2. Sample Size Calculation

Sample size was estimated with a simulation-based power analysis for changes in maximal isometric voluntary contractions (MVC) using the ANOVA_power app [25]. The analysis suggested with a change in means and SD of 10 and 8%, respectively, and 12 participants per group, we had ≥85% power to detect an interaction effect (effect size of 0.20 (partial eta squared; ηp^2^) at 24–72 h post-exercise. The mean and SD for the analysis were estimated from similarly designed studies examining interventions on recovery of MVC from muscle damaging exercise [26,27]. 

### 2.3. Experimental Design

Ethical approval was granted by the Loughborough University Research Ethics Committee (ethics approval number: 15763) and all procedures were conducted in accordance with the Declaration of Helsinki (1964). The protocol was pre-registered on the Clinical Trials registry, the Open Science Framework (Project Registration Link: https://osf.io/qgw3a/, accessed on 18 February 2026 and registration number: qgw3a; 25 October 2023), and the study employed a double blind, independent groups design with two experimental conditions. On participants’ first visit, height, mass, maximal isometric voluntary contractions (MVC) and maximal countermovement jump (CMJ) height were collected. Participants were also familiarised with the techniques to be utilised to record reactive strength index (RSI), the drop jump exercise protocol, and pressure pain threshold (PPT). Participants were then randomised according to their sex and MVC, using the minimisation method to ensure baseline equality between the conditions. Randomisation was performed by an individual not involved in data collection; all trial researchers were blinded to the conditions until after statistical analysis was completed. After this visit participants were provided with their interventions along with instructions to consume them upon waking for 6 days prior to their second visit.

On the second visit to the laboratory participants performed the bout of muscle damaging exercise. Before exercise, primary and secondary outcomes were collected in the following order: resting blood and urine samples, subjective sleep quality, subjective exhaustion (Hecimoviche-Peiffere-Harbough-Exercise Exhaustion survey (HPHEE), muscle soreness, PPT, MVC, CMJ, RSI. Once all outcomes were recorded, participants consumed a cereal bar (Nature Valley Honey and Oat cereal bar, 42 g, General Mills International, Sárl, Nyon, Switzerland) and rested for 30 min before performing a strenuous plyometric exercise protocol designed to induce muscle damage. Further blood and urine samples were collected immediately post exercise and 2.5 h post-exercise. One dose of their supplement was consumed immediately after the post-exercise time-point. Between the post-exercise and 2.5 h post-exercise samples, participants rested and consumed another cereal bar with 500 mL of water. Participants returned to the laboratory on consecutive days for 3 more visits corresponding to 24, 48 and 72 h post-exercise. At each visit, the same outcomes collected pre-exercise were recorded in the same order. All visits took place after a ≥10 h fast, with supplements consumed after outcomes were recorded on the 24 and 48 h post-exercise visits.

### 2.4. Supplementation

Participants were randomly allocated to consume either the phytochemical blend (PB) or placebo control (CON) supplement for 6 days prior to exercise, on the day of exercise, and for two days following exercise (9 days in total). In the 6 days prior to the exercise trial, participants consumed one 300 mg capsule upon waking; on the day of exercise, this capsule was consumed immediately after exercise, and at 24 and 48 h post-exercise it was consumed after the dependent variables were collected. The intervention was provided 6 days prior to the exercise bout based on previous research suggesting ‘pre-loading’ with phytochemicals could be more efficacious than single acute doses [28].

The PB supplement dose was formulated to contain 108 mg of calcium fructoborate along with 96 mg of *Curcuma longa* extract standardised to ≥95% curcuminoids and 96 mg of *Punica granatum* extract standardised to ≥40% punicalagins (VDF FutureCeuticals, Momence, IL, USA). The CON supplement dose was a microcrystalline cellulose powder (Sigma Aldrich, St. Louis, MO, USA). The supplements were identical in appearance and provided to participants in the same opaque bottles.

Throughout the trial, participants were told to maintain their habitual diet, but to avoid any increase in consumption of (poly)phenol rich foods. To ensure adherence, a list of common foods high in (poly)phenols was provided to participants alongside a 3-day food diary, whereby participants were told to record all food and beverages consumed immediately following visit 2 (main exercise trial) and on the following 2 days. These were visually inspected for adherence and group disparities.

### 2.5. Muscle Damaging Exercise

Muscle damage was induced with a plyometric-based drop jump protocol [29]. Participants performed a total of 150 drop jumps from a 60 cm box in sets of 6 × 25 repetitions. Each set was separated by a 2 min rest period and each jump by 10 s. For each jump, participants landed on a contact jump mat (JumpMat™, FSL Scoreboards, Cookstown, Northern Ireland) with 2 feet before squatting to a ~90° knee angle and then jumping vertically with maximal effort. Verbal guidance and encouragement was provided throughout to ensure participants reached >80% of the maximal jump height recorded at familiarisation.

### 2.6. Markers of Muscle Soreness

Muscle soreness was measured subjectively with a visual analogue scale (VAS) and PPT, as per previously described methods [30]. To measure subjective muscle soreness, participants performed a squat (at ~90° knee flexion) and rated their lower limb muscle soreness by drawing a vertical line on a VAS anchored by 0 (‘no soreness’) and 200 mm (‘unbearably painful’). The line placement was measured with a ruler and recorded. PPT was measured with a handheld algometer (Wagner Instruments, Greenwich, CT, USA). With the participant lying supine, a cylindrical flat headed probe (1 cm diameter) was used to apply pressure to a pre-marked site on the muscle belly until the participant signified that they felt pain or discomfort, at which point a force value was recorded (N/cm^2^). PPT was measured in three muscles: vastus lateralis, mid-way between the superior aspect of the greater trochanter and the tibia head; rectus femoris, mid-way between the anterior patella and inguinal fold; gastrocnemius, medial aspect of the calf muscles at its relaxed maximum girth. All measures were taken by the same individual and on the participant’s dominant leg. Each site was measured twice, with a third taken if the measures differed by >10 N/cm^2^. An average of the closest 2 recordings was used for analysis. Using this procedure we have calculated inter-day coefficient of variation (CV) for PPT measures as ≤4.9%.

### 2.7. Maximal Isometric Voluntary Contractions

MVC was measured in newtons of force using a custom-built rig [31]. Each contraction was performed seated upright. Participants were firmly strapped across the waist and torso into the chair to limit movement, with the chair adjusted to align to the individual participants stature; these height adjustments were recorded and consistent for each participant at each measurement. A strain gauge was fitted and tightened to their dominant foot, located just above the malleoli. At each time point, participants performed 3 submaximal contractions corresponding to 50, 75, and 90% of perceived maximal effort, each separated by 30 s rest. Thereafter, participants performed 3 MVCs, each separated by 60 s rest. For each maximal contraction, participants were instructed to exert maximal force by extending their knee and holding an isometric contraction for 3 s; strong verbal encouragement was provided during these efforts. Force data was recorded and analysed in Spike2 version 6 software (CED, Cambridge, UK). The average of the 3 MVC efforts were calculated and used for analysis. The inter-day CV for MVC measures was ≤4.8%.

### 2.8. Countermovement Jump and Reactive Strength Index

CMJ and RSI metrics were calculated with the MyJumpLab Application (version 3.1.1) [32] using previously validated methods [33]. To record the jumps, videos were taken with an IPAD placed ~0.75 m from the floor and ~1.5 m from the participants feet. Jumps were recorded in the RSI_mod function. For the CMJ, participants stood with their feet shoulder width apart, descended into a squat (to a ~90º knee angle) before rebounding with a vertical jump; hands were fixed to their hips throughout the whole movement. The beginning, take-off, and landing phase were isolated from the recording to calculate height (cm), time to take-off (ms), RSI_mod (AU), and flight time (ms). For RSI, participants dropped from a 30 cm box and, upon landing, immediately jump vertically, with instructions to minimise ground contact time while maximising jump height [29]. The first contact, take-off, and landing contact phases were isolated from the recording to calculate height (cm), flight time (ms) and contact time (ms). RSI was calculated as jump height/contact time. For both CMJ and RSI, three maximal efforts were performed, separated by 60 s of passive (standing) recovery. Several studies have shown that MyJumpLab is a reliable and valid tool to measure CMJ and RSI metrics [33,34,35]. The inter-day CV for this analysis was ≤5.1%.

### 2.9. Measures of Perceived Sleep Quality and Exhaustion

To explore the effects of the intervention on subjective exhaustion, participants completed the HPHEE survey [36] pre-exercise, and 24 h, 48 h, and 72 h post-exercise. This survey consists of 14 items related to exercise exhaustion, such as “How much do your muscles ache” and “How mentally drained do you feel”. Each item was evaluated on a 1 (not at all/not difficult) to 10 (extremely/extremely difficult) Likert-scale; higher scores were negative. The average and individual responses to each of the 14 items were analysed. At the same time-points, perceived sleep quality was recorded as described previously [37]. Participants rated their sleep quality on a 1 (very poor)–4 (very good) Likert scale.

### 2.10. Biological Sample Collection

At each time point (pre-exercise, post-exercise, 2.5, 24, 48, and 72 h post-exercise), venous blood was drawn via venepuncture into tubes treated with either ethylenediaminetetraacetic acid (EDTA) (for full blood counts and plasma collection), or for serum collection. Serum tubes were left to clot for 30 min at room temperature without agitation, while EDTA blood was immediately processed to measure full blood counts using a Yumizen H500 cell counter (Horiba Medical, Montpellier, France). EDTA and serum tubes were then centrifuged at 1500× *g* for 10 min at 4 °C. Isolated plasma and serum were subsequently pipetted into duplicate cryovials and stored at −80 °C for later analysis. At the same time points, urine was collected into a 50 mL tube then aspirated into a 1.5 mL Eppendorf tube containing 0.005% 2,6-di-tert-butyl-4-methylphenol in ethanol (for isoprostane analysis) and stored at −80 °C for later analysis.

### 2.11. Biological Markers of Inflammation, Muscle Damage, and Oxidative Stress

Total leukocytes, neutrophils, lymphocytes, monocytes, and platelets, were measured with a Yumizen H500 cell counter (Horiba Medical, Montpellier, France). Creatine kinase (CK), lactate dehydrogenase (LDH) and high sensitivity c-reactive protein (hs-CRP) were measured photometrically in serum using an automated system (Roche Cobas c720, Roche Diagnostics, Burgess Hill, UK). Commercially available enzyme linked immunosorbent assays (ELISA) were used to measure plasma concentrations of interluekin-6 (IL-6) (Human IL-6 Quantikine HS ELISA HS600C, R&D Systems, Oxford, UK), monocyte chemoattractant protein-1 (MCP-1) (Human CCL2/MCP-1 ELISA Kit, R&D Systems, Oxford, UK), and protein carbonyls (PC) (Redox Innovation, University of Otago, Christchurch, New Zealand). Urinary concentrations of isoprostanes (free 15-isoprostane F_2t_) were also measured with an ELISA (Urinary Isoprostane, Oxford Biomedical Research, EA85.5, Oxford, UK) and are presented corrected for creatinine concentrations (Creatinine Colorimetric Assay Kit, 500701, Cayman Chemical, Ann Arbor, MI, USA). All analysis was performed according to the manufacturer’s instructions; intra-plate CVs for this analysis were ≤7.8%.

### 2.12. Data Analysis

Participant physical characteristics and exercise intensity during the drop jumps were analysed with independent *t*-tests. Muscle function, subjective muscle soreness, and subjective sleep and exhaustion scales were measured with mixed model ANOVAs with 2 between factor conditions (PB and CON) and 4 repeated measures time-points (pre-exercise, 24 h, 48 h, and 72 h post-exercise). For PPT, pre-exercise values were added as a covariate to account for the large between group imbalances (~20% difference). Normal distribution of data was confirmed with the Shapiro–Wilk test (*p* < 0.05 not normally distributed) and by inspecting Q-Q plots of the residuals; homogeneity of variance was confirmed by Levene’s test (if *p* > 0.05 than homogeneity assumed). Across our analysis, Levene’s test was F ≤ 3.326; *p* ≥ 0.076 and therefore homogeneity of variance assumed. If Mauchly’s test of sphericity was *p* < 0.05, the Greenhouse–Geisser or Huynd–Feldt (if ε is greater than 0.75) adjusted *p* value is presented. Normality was assumed for CMJ time to take off (CMJ_TTO) after log10 transformation. Biological samples were measured with linear mixed models to account for the 4 samples missing at random. For mixed effects analysis, participants were added as random effects, and time and condition were added as fixed effects. Normality was confirmed with the Shapiro–Wilk test, histograms and Q-Q plots of the residuals; homogeneity of variance was confirmed by plotting the residuals vs. the predicted values. If raw data were not normally distributed, they were transformed using Log10 or Box–Cox and normality was reevaluated. CK, monocytes, and MCP-1 were normally distributed after log10 transformation. While transformation successfully reduced the skew of all the relevant variables, the Shapiro–Wilk test was still highly statistically significant (*p* < 0.05) for some variables (LDH, platelets, hs-CRP, PC, isoprostanes and IL-6) and therefore these data were analysed with a generalised linear mixed model (GLMM) using a gamma distribution and link log function. This analysis did not change the statistical outcomes and interpretations for any of these variables. On account of this being the first time this intervention has been examined in this context, *post hoc* tests were performed with Fisher’s least significant differences method. Subjective sleep (PSQ) and exhaustion (HPHEE) were measured with both mixed model ANOVAs and, on account of being ordinal data, with non-parametric Mann–Whitney U tests, as described previously [38]. For completeness, statistical analysis using both methods is presented. Each item on the HPHEE was also analysed individually. *p* < 0.05 was considered statistically significant. Partial eta squared effect sizes (ηp^2^) were added for main ANOVA effects. For consistency and comparability, we also calculated ηp^2^ effect sizes for LMM analysis; however, these only explain the variation in the fixed effects and do not account for random effects and thus should be interpreted accordingly. We have reported the conditional R^2^ values to account for the overall variance for the LMM and GLMMs. ηp^2^ were added for main effects: 0.010–0.06 was considered a small effect; 0.06–0.14 a medium effect; ≥0.14 a large effect [39]. Cohens *d_s_* effect sizes were added for 2 sample analysis (0.20–0.49 was considered a small effect; 0.49–0.79 a medium effect; >0.80 a large effect [39]. For non-parametric analysis, rank biserial correlations (*r*_rb_) are also presented to estimate effect sizes; these fall within 0 and 1, the higher number indicates a larger effect. All data analysis was completed on jamovi (The jamovi project (2024). *jamovi* (Version 2.6) [Computer Software]. Retrieved from https://www.jamovi.org).

## 3. Results

There was 100% compliance with the supplementation, and dietary and exercise restrictions and no adverse events were reported. There were no differences in participants’ physical characteristics or their jump height during the exercise protocol (Table 1).

### 3.1. Neuromuscular Performance

Neuromuscular performance data is presented in Table 2. Except for CMJ_TTO, there was a statistically significant decrease in all markers of neuromuscular performance after exercise (*p* < 0.05) but there were no condition or interaction effects.

### 3.2. Pressure Pain Threshold and Subjective Muscle Soreness

**Figure 2:**
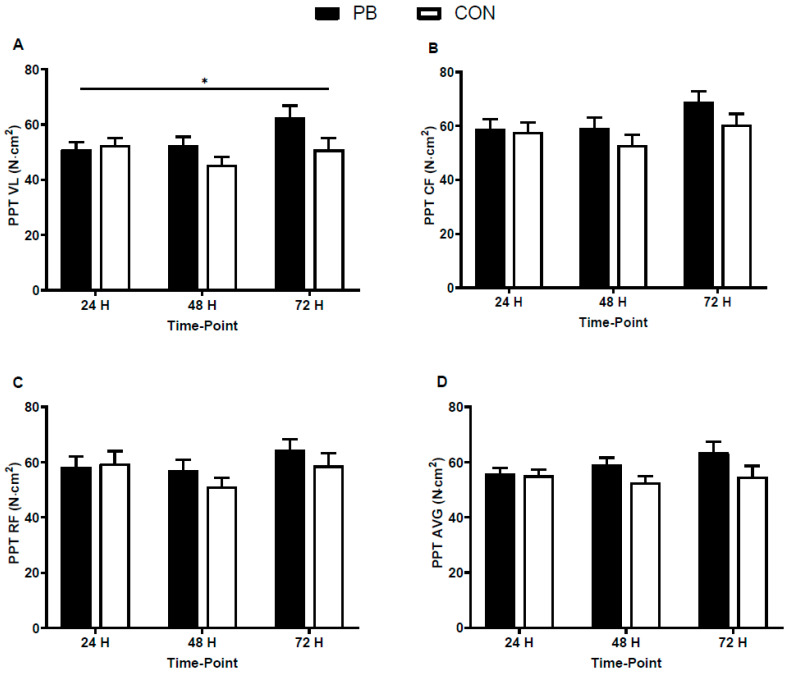
Pressure pain threshold (PPT) in the two supplement conditions (PB, phytochemical blend, CON, control). Data presented for (**A**) vastus lateralis (VL); (**B**) calf (CF); (**C**) rectus femoris (RF); (**D**) average. * Indicates time x condition interaction effect (*p* < 0.05). Data presented represents values at 24, 48 and 72 h post-exercise; pre values were added as a covariate. Values presented are the adjusted estimated marginal means and standard errors.

Pressure pain threshold data are presented in Figure 2. For the average of all three muscle sites, there was no condition (F = 2.02; *p* = 0.170; ηp^2^ = 0.088) or interaction effects (F = 2.77; *p* = 0.105; ηp^2^ = 0.116). There was no condition effect for vastus lateralis PPT (F = 2.06; *p* = 0.166; ηp^2^ = 0.088) but there was an interaction effect (F = 3.98; *p* = 0.041; ηp^2^ = 0.159) for higher PPT in PB vs. CON. *Post hoc* tests did not reveal statistically significant differences at 24 h (*p* = 0.731; *d_s_* = 0.141), 48 h (*p* = 0.117; *d_s_* = 0.677) or 72 h post-exercise (*p* = 0.074; *d_s_* = 0.767). There was no condition (F = 1.25; *p* = 0.276; ηp^2^ = 0.056) or interaction effects (F = 1.136; *p* = 0.321; ηp^2^ = 0.051) for calf PPT, and rectus femoris PPT (condition, F = 0.558; *p* = 0.463; ηp^2^ = 0.026), (interaction, F = 1.19; *p* = 0.300; ηp^2^ = 0.054).

Subjective muscles soreness increased after exercise in both conditions (time effect; F = 65.259; *p* < 0.001; ηp^2^ = 0.748) (PB vs. CON: pre-exercise = 14 ± 9 vs. 13 ± 16 mm; 24 h post-exercise = 127 ± 37 vs. 119 ± 46 mm; 48 h-post-exercise = 118 ± 47 vs. 107 ± 64 mm; 72 h-post-exercise = 68 ± 44 vs. 54 ± 49 mm. However, there were no condition (F = 0.378; *p* = 0.545; ηp^2^ = 0.017) or interaction effects (F = 0.175; *p* = 0.913; ηp^2^ = 0.008).

### 3.3. Perceived Sleep Quality and Exhaustion

The PSQ and HPHEE data is displayed in Table 3. For PSQ, the ANOVA analysis showed no time (F = 0.166; *p* = 0.871; ηp^2^ = 0.007), condition (F = 3.10; *p* = 0.092; ηp^2^ = 0.123) or interaction effects (F = 1.373; *p* = 0.263; ηp^2^ = 0.059). The HPHEE increased after exercise (time effect; F = 26.83; *p* < 0.001; ηp^2^ = 0.549) but there was no condition (F = 0.559; *p* = 0.462; ηp^2^ = 0.025) or interaction effects (F = 2.00; *p* = 0.122; ηp^2^ = 0.083). In our exploratory analysis, two of the items on the HPHEE showed condition or interaction effects. There was an interaction effect for “How recovered do you feel” (F = 2.86; *p* = 0.043; ηp^2^ = 0.115), with *post hoc* tests showing that recovery was rated better in PB vs. CON at 24 h (5.2 ± 2.0 vs. 7.2 ± 1.0) and 48 h (4.8 ± 2.1 vs. 6.3 ± 1.2) post-exercise (*p* ≤ 0.034; *d_s_* ≥ 0.92). There was also a condition effect for “How mentally drained do you feel” (F = 4.86; *p* = 0.038; ηp^2^ = 0.181). It should be noted that these variables were ordinal and showed some deviations from normality and therefore should be interpreted accordingly. Hence, these are complimented with non-parametric analysis.

Mann–Whitney U tests were performed to examine condition differences at each time-point, as well as an average score from 24 to 72 h post-exercise. PSQ was higher in PB vs. CON at 72 h-post-exercise (*p* = 0.049; *r*_rb_ = 0.423) and 24–72 h post-exercise *p* = 0.032; *r*_rb_ = 0.458. There were no significant differences in the HPHEE at any time points; however, for the individual item, “How recovered do you feel”, scores were rated better in PB vs. CON at 24 h (*p* = 0.004; *r*_rb_ = 0.694), 48 h (*p* = 0.035; *r*_rb_ = 0.500) post-exercise, and for 24–72 h-postexercise average (*p* = 0.023; *r*_rb_ = 0.542); For the item “How mentally drained do you feel”, PB rated better than CON at 72 h post-exercise (*p* = 0.044; *r*_rb_ = 0.472) and for the 24–72 h-postexercise average (*p* = 0.030; *r*_rb_ = 0.500).

### 3.4. Blood and Urine Sample Analysis

Full blood count data is presented in Table 4, with biomarkers of muscle damage, inflammation, and oxidative stress presented in Table 5. Apart from isoprostanes, all biomarkers increased after exercise (*p* > 0.05) but there were no significant interaction effects for any marker (*p* > 0.05). As there were large pre-exercise differences in PC and hs-CRP, to check the robustness of the findings they were also analysed with pre-exercise values added as a covariate (these data assumed normal distribution according to methods outlined in Section 2.12). This did not change interpretation of the statistical analysis for hs-CRP (F ≤ 0.195; *p* ≥ 0.05) but did for PC; there was no longer a condition or interaction effect (both F ≤ 3.01; *p* ≥0.087; ηp^2^ ≤ 0.150) suggesting the pre-exercise differences were partly driving this effect.

## 4. Discussion

The main finding of this study is that a multi-ingredient phytochemical supplement tended to reduce muscle soreness, as measured by pressure pain threshold, after muscle-damaging exercise. Supplementation did not favourably affect the recovery of muscle function, biomarkers of muscle damage, inflammation, or oxidative stress, but perceived sleep quality was higher, and some aspects of perceived exhaustion were lower in the days after exercise. Interestingly, our data suggests that while the intervention may not affect objective aspects of recovery like muscle function, it may enhance the perceptual feelings of recovery. This is the first study to examine the effects of this unique blend of phytochemicals on markers of exercise-induced muscle damage and suggests that the supplement holds promise as a strategy to attenuate muscle soreness and support perceived recovery after muscle damaging exercise.

We found no differences in subjective muscle soreness, as measured with a VAS, but the PPT at the vastus lateralis was significantly higher in the PB vs. CON group post-exercise, suggesting lower muscle soreness. The changes were not statistically significant with *post hoc* tests, but medium effect sizes for differences were found at 48 and 72 h post-exercise (*d*_s_ = ≥0.67). There was also a medium effect size for higher PPT in the calf at 72 h post, and the average PPT across all three muscle groups, suggesting a general pattern for reduced muscle soreness in the intervention groups. Such effect sizes are in line with a recent meta-analysis that suggested phytochemical supplements have small to medium effects on muscle soreness after exercise [21]. Although the effect sizes are not large, they could be meaningful, especially in athletic populations, where fine margins are important. Indeed, previous research suggests that a change of 20% in PPT, as seen in the VL in the present study, could be considered clinically meaningful [40]. The increase in PPT somewhat agrees with other studies that provided the phytochemicals in our combined supplement; indeed, curcumin and pomegranate have both been shown to attenuate exercise-induced muscle soreness in doses up to 5 g/day [15,16,41]. However, findings are inconsistent, with other studies reporting no effects on muscle soreness after pomegranate [42,43] or curcumin supplementation [44]. Recent meta-analyses on the topic [20,42] suggested that the equivocal findings amongst studies are likely because of the different doses used and extent of muscle damage, which are compounded by small effect sizes. Short-term and long-term supplementation with calcium fructoborate has previously been shown to reduce joint and muscle pain in non-exercising persons [17,18]. This is the first study, however, to examine the effects of calcium fructoborate after strenuous exercise.

It is not completely understood how exercise elicits muscle soreness, but inflammation and oxidative stress are thought to play a role in its development [45]. Indeed, recent animal studies have shown that blocking the expression of cytokines such as IL-6 and TNF-alpha [46] and the cyclooxygenase pathway [47] can reduce muscle soreness, likely by reducing the expression of neurotrophins that sensitise nociceptors [48]. In the absence of muscle biopsies, we measured inflammatory markers, as well as lipid and protein oxidation in urine and blood, respectively, but found no evidence of the intervention reducing inflammation or oxidative stress post-exercise. Our findings are broadly in line with recent meta-analyses, which have found limited and inconsistent evidence of (poly)phenols attenuating systemic indices of inflammation and oxidative stress, often despite a general pattern for reduced muscle soreness [20,42]. It is possible that the changes in muscle soreness in this study and others were the result of local changes in the skeletal muscle and surrounding extracellular matrix, where muscle soreness may originate and predominate [49]. However, this is speculative, as we did not measure changes in muscle structure, and few studies have correlated changes in muscle soreness and inflammation locally [12] to support this. It was beyond the scope of this study, however, to directly analyse the mechanisms behind the reduced muscle soreness, but this should be explored more in future research.

Neuromuscular performance, measured isometrically as maximum strength, or dynamically via CMJs and drop jumps, were unaffected by supplementation. The effects of various phytochemicals, including those in the supplement we provided, have shown inconsistent effects on markers of neuromuscular performance. While individual studies have shown potential benefits for strength recovery after supplementation with curcumin [50] and pomegranate [15], recent meta-analyses of human trials with these supplements have not reported overall favourable effects on recovery of neuromuscular performance [20,42], irrespective of dose. Several factors are suggested to contribute to these inconsistent effects, including the wide range of doses employed and the methods used to induce muscle damage and assess neuromuscular function and poor study design (e.g., inadequate blinding, use of unvalidated neuromuscular methodologies). While higher doses may yet prove more effective, there is a need for more high-quality studies to adequately explore the application of phytochemical supplements (in single and multi-component formulations) for the amelioration of post EIMD muscle soreness as well as neuromuscular deficits.

Given that inflammation [51] and pain [52] may negatively affect sleep, and, in some studies, phytochemicals may improve sleep [53,54], we included a measure of subjective sleep quality as an exploratory outcome. Interestingly, PSQ was higher in the intervention group in the days after exercise. The mechanisms underlying these findings are unclear. It seems unlikely that the supplement directly influenced sleep as they were consumed 12–15 h before sleep, at which point the bioactive metabolites would likely be excreted. Rather, the effects may have been indirect and associated with the PPT results. Musculoskeletal pain and sleep are closely associated [55,56] and are thought to have a bidirectional relationship, with pain negatively impacting sleep quality, and poor sleep exacerbating the painful symptoms [52]. While the causal direction of this relationship remains debated, it is plausible that in our study the greater PSQ after the intervention was, at least in part, due to participants feeling less muscle soreness. The reduced muscle soreness could also partly explain why the participants reported feeling more recovered and less mentally drained (subjectively) after the exercise with the intervention. Explanations for our findings are presently speculative, however, and require additional confirmatory studies with similarly valid and objective markers of sleep quality and subjective exhaustion. Nevertheless, we are unaware of any other reports of a short-term nutritional intervention supporting post-exercise recovery through reduced muscle soreness and improved perceptions of sleep quality, recovery and mental fatigue.

This study has limitations to acknowledge. Firstly, we did not quantify the plasma concentrations of the specific phytochemicals in the supplement, or their metabolites. Thus, we do not know for certain that the phytochemical concentrations were significantly elevated in the intervention group. Nor can we positively establish that the effects observed here, in a dose markedly lower than typical curcumin and pomegranate interventions, are not the result of some complementary or synergistic action. Furthermore, we were unable to obtain muscle biopsies to assess the local inflammatory response to the supplement. Future studies may also benefit from including direct but non-invasive measures of tissue damage, such as with the use of magnetic resonance imaging. Directly interrogating the underlying mechanisms of the supplements biochemical effects was beyond the scope if this study; indeed, our focus was the functional consequences of changes in muscle soreness and neuromuscular performance. However, as we only measured inflammation and oxidative stress in the circulation, we cannot rule out any anti-inflammatory or antioxidant effect in the skeletal muscle or other tissues. Strengths of this study include the randomised placebo-controlled study design, the comprehensive battery of EIMD markers, the highly characterised nature of the supplement and the use of a dosage strategy (one capsule per day) that is practical and realistic for consumers.

## 5. Conclusions

In conclusion, this is the first study to examine the effects of a novel phytochemical blend, at a relatively low dose, on markers of EIMD, and suggests that the supplement holds promise as a recovery strategy to attenuate muscle soreness and perceived exhaustion after exercise. In particular, the supplement appeared to enhance the perceptual aspects of recovery. The mechanisms by which this unique blend of ingredients may have affected these aspects of recovery, and enhanced perceived sleep quality, warrant further investigation.

## Figures and Tables

**Table 1:** Physical characteristics and jump heights during the exercise protocol for participants in the phytochemical blend (PB) and control (CON) groups.

	CON (5 Females, 7 Males)	PB (5 Females, 7 Males)	*p* Value
**Age (years)**	24 ± 4	23 ± 2	0.424
**Body mass (kg)**	70.7 ± 11.5	71.7 ± 17.3	0.865
**Height (cm)**	170.9 ± 11.3	171.6 ± 10.7	0.894
**Average jump height (cm)**	28.9 ± 7.8	28.3 ± 6.8	0.838
**Difference from maximum jump height (%)**	−14 ± 9	−15 ± 9	0.670

**Table 2:** Markers of neuromuscular performance in the phytochemical blend (PB) and control (CON) conditions pre-exercise to 72 h post-exercise.

	**Pre**	**24 h**	**48 h**	**72 h**	**Time; Condition; Interaction**	ηp^2^: Time; Condition; Interaction
**MVC (N** **·** **kg** **·** **bm^−1^)**						
** CON**	10.4 ± 1.9	8.7 ± 2.3	9.3 ± 2.4	9.8 ± 2.5	F = 15.576; F = 0.175; F = 0.380 *p* < 0.001; *p* = 0.680; *p* = 0.635	0.415; 0.008; 0.017
** PB**	10.4 ± 1.6	8.4 ± 1.8	8.8 ± 1.9	9.3 ± 2.0		
**CMJ (cm)**						
** CON**	30.7 ± 8.0	28.2 ± 8.8	27.0 ± 8.9	28.0 ± 9.1	F = 18.74; F = 0.129; F = 0.113 *p* < 0.001; *p* = 0.723; *p* = 0.343	0.460; 0.006; 0.049
** PB**	31.0 ± 6.7	26.4 ± 7.0	25.1 ± 8.2	26.7 ± 8.9		
**CMJ_TTO (ms)**						
** CON**	841 ± 159	933 ± 236	931 ± 206	925 ± 180	F = 2.86; F = 0.52; F = 1.04 *p* = 0.052; *p* = 0.822; *p* = 0.376	0.115; 0.045; 0.002
** PB**	867 ± 158	928 ± 182	917 ± 185	857 ± 189		
**CMJ_FT (ms)**						
** CON**	499 ± 71	473 ± 76	463 ± 79	470 ± 83	F = 14.737; F = 0.141; F = 0.462 *p* < 0.001; *p* = 0.711; *p* = 0.667	0.401; 0.006; 0.021
** PB**	497 ± 56	458 ± 67	448 ± 79	458 ± 77		
**RSI_MOD (AU)**						
** CON**	0.38 ± 0.14	0.33 ± 0.14	0.32 ± 0.15	0.33 ± 0.15	F = 10.72; F = 0.230; F = 0.741 *p* < 0.001; *p* = 0.636; *p* = 0.531	0.318; 0.010; 0.033
** PB**	0.37 ± 0.10	0.29 ± 0.80	0.28 ± 0.08	0.32 ± 0.10		
**RSI (AU)**						
** CON**	1.77 ± 0.41	1.53 ± 0.59	1.52 ± 0.50	1.66 ± 0.52	F = 13.37; F = 0.1.01; F = 0.193 *p* < 0.001; *p* = 0.325; *p* = 0.880	0.378; 0.044; 0.009
** PB**	1.64 ± 0.42	1.32 ± 0.35	1.33 ± 0.38	1.50 ± 0.42		
**RSI_FT (ms)**						
** CON**	445 ± 54	486 ± 75	398 ± 66	406 ± 68	F = 16.679; F = 0.864; F = 0.946 *p* < 0.001; *p* = 0.363; *p* = 0.409	0.431; 0.038; 0.041
** PB**	434 ± 57	383 ± 83	361 ± 93	385 ± 87		
**RSI_CT (ms)**						
** CON**	264 ± 53	294 ± 66	280 ± 68	261 ± 59	F = 4.435; F = 0.130; F = 0.081 *p* = 0.015; *p* = 0.722; *p* = 0.933	0.168; 0.006; 0.004
** PB**	278 ± 59	304 ± 96	285 ± 79	269 ± 67		

AU, arbitrary units; CMJ, countermovement jump height; CT, contact time; FT, flight time; MVC, maximal isometric voluntary contraction; MOD, modified; ms milliseconds; N, Newtons; RSI, reactive strength index; TTO, time to take off. ηp^2^ = partial eta squared. Pre = pre-exercise. Data are means ± standard deviation.

**Table 3:** Perceived sleep quality (PSQ) and Hecimoviche–Peiffere–Harbough Exercise Exhaustion survey (HPHEE) pre—72 h post-exercise in the control (CON) and phytochemical blend (PB) conditions.

	Pre	24 h	48 h	72 h	24–72 h #
**PSQ**					
**CON**					
** ** **Mean ± SD**	3.1 ± 0.5	3.1 ± 0.3	2.8 ± 0.7	2.9 ± 0.5	3.0 ± 0.4
** ** **Median (IQR)**	3.0 (0.0)	3.0 (0.0)	3.0 (0.0)	3.0 (0.0)	3.0 (0.0)
**PB**					
** ** **Mean ± SD**	3.2 ± 0.8	3.3 ± 0.7	3.4 ± 0.8	3.4 ± 0.7	3.5 ± 0.7
** ** **Median (IQR)**	3.0 (1.0)	3.0 (1.0)	4.0 (1.0)	3.5 (1.0) *	4.0 (1.0) *
**HPHEE**					
**CON**					
** ** **Mean ± SD**	2.2 ± 0.8	5.2 ± 1.1	5.2 ± 0.8	3.5 ± 1.7	4.7 ± 1.0
** ** **Median (IQR)**	2.0 (0.3)	5.0 (2.0)	5.0 (1.0)	3.0 (2.3)	4.0 (2.0)
**PB**					
** ** **Mean ± SD**	2.8 ± 1.6	4.4 ± 1.1	4.6 ± 1.8	3.2 ± 1.4	4.0 ± 1.3
** ** **Median (IQR)**	2.5 (1.3)	4.5 (1.3)	5.0 (3.0)	3.0 (1.3)	4.0 (2.0)

24–72 h = average score at these time-points. * *p* < 0.05 Mann–Whitney U tests. # average score 24–72 h post-exercise.

**Table 4:** Leukocytes, neutrophils, lymphocytes, and platelets pre-72 h post-exercise in the control (CON) and phytochemical blend (PB) groups.

	Pre	Post	2.5 h	24 h	48 h	72 h	Time; Condition; Interaction	ηp^2^ Time; Condition; Interaction (R^2^)
**Leukocytes (10^9^/L)**								
** CON**	5.49 ± 0.89	7.30 ± 1.53	6.90 ± 0.98	5.41 ± 0.96	5.15 ± 0.84	5.37 ± 1.09	F = 31.70; F = 0.165; F = 0.098 *p* < 0.001; *p* = 0.688; *p* = 0.992	0.509; 0.006; 0.004 (0.829)
** PB**	5.77 ± 1.91	7.47 ± 2.65	7.15 ± 2.31	5.86 ± 2.03	5.29 ± 1.66	5.68 ± 1.81		
**Neutrophils (10^9^/L)**								
** CON**	2.86 ± 0.61	3.94 ± 0.99	4.47 ± 0.94	2.88 ± 0.55	2.57 ± 0.54	2.82 ± 0.56	F = 44.32; F = 0.072; F = 0.032 *p* < 0.001; *p* = 0.791; *p* = 0.999	0.676; 0.001; 0.002 (0.844)
** PB**	2.82 ± 1.41	3.80 ± 1.82	4.37 ± 1.86	2.81 ± 1.50	2.50 ± 1.15	2.69 ± 1.03		
**Lymphocytes (10^9^/L)**								
** CON**	1.94 ± 0.45	2.54 ± 0.69	1.73 ± 0.36	1.86 ± 0.54	1.90 ± 0.48	1.90 ± 0.61	F = 5.61; F = 2.07; F = 1.18 *p* < 0.001; *p* = 0.164; *p* = 0.326	0.209; 0.086; 0.052 (0.635)
** PB**	2.20 ± 0.59	2.82 ± 0.84	2.03 ± 0.51	2.32 ± 0.62	2.08 ± 0.53	2.27 ± 0.74		
**Monocytes (10^9^/L)**								
** CON**	0.42 ± 0.10	0.55 ± 0.15	0.48 ± 0.12	0.40 ± 0.16	0.39 ± 0.13	0.41 ± 0.16	F = 9.745; F = 1.202; F = 0.415 *p* < 0.001; *p* = 0.285; *p* = 0.837	0.314; 0.051; 0.019 (0.720)
** PB**	0.49 ± 0.16	0.57 ± 0.18	0.53 ± 0.15	0.47 ± 0.12	0.45 ± 0.16	0.47 ± 0.12		
**Platelets (10^9^/L) †**								
** CON**	246 ± 40	296 ± 50	259 ± 40	251 ± 44	258 ± 52	265 ± 46	χ^2^= 63.55; χ^2^ < 0.001; χ^2^ = 3.32 *p* < 0.001; *p* = 0.990; *p* = 0.651	(0.622)
** PB**	263 ± 80	309 ± 110	282 ± 100	275 ± 87	259 ± 92	279 ± 91		

ηp^2^ = partial eta squared, presented for linear mixed model (LMM) analysis only. Pre = pre-exercise; Post = post-exercise. R^2^ = conditional R^2^ for LMM and generalised linear mixed model (GLMM). † GLMM analysis performed as data not normally distributed. χ^2^ statistic presented for GLMM analysis. Data are means ± standard deviation.

**Table 5:** Systemic biomarkers of inflammation, muscle damage, and oxidative stress phytochemical blend (PB) and control (CON) groups pre-exercise to 72 h post-exercise.

	Pre	Post	2.5 h	24 h	48 h	72 h	Time; Condition; Interaction	ηp^2^ Time; Condition; Interaction (R^2^)
**Hs-CRP (pg/mL) †**								
** ** **CON**	1.60 ± 3.28	1.72 ± 3.60	1.73 ± 3.67	1.66 ± 2.87	1.43 ± 1.62	1.22 ± 1.18	χ^2^ = 13.95; χ^2^ = 0.846; χ^2^ = 0.868 *p* = 0.016; *p* = 0.358; *p* = 0.973	(0.689)
** ** **PB**	0.86 ± 1.01	0.85 ± 1.07	0.85 ± 0.97	0.94 ± 1.12	1.21 ± 1.77	1.07 ± 1.55		
**IL-6 (pg/mL) †**								
** ** **CON**	0.74 ± 0.49	0.97 ± 0.52	1.06 ± 0.81	0.65 ± 0.38	0.53 ± 0.41	0.56 ± 0.33	χ^2^ = 81.88; χ^2^ = 0.04; χ^2^ = 2.88 *p* < 0.001; *p* = 0.829; *p* = 0.717	(0.541)
** ** **PB**	0.81 ± 0.68	1.03 ± 0.97	1.23 ± 0.42	0.62 ± 0.24	0.66 ± 0.41	0.58 ± 0.28		
**MCP-1 (pg/mL)**								
** ** **CON**	148.9 ± 29.3	165.0 ± 39.7	155.6 ± 39.9	155.7 ± 33.2	147.9 ± 52.4	151.0 ± 46.7	F = 3.95; F = 0.075; F = 1.121 *p* = 0.003; *p* =0.786 *p* = 0.306	0.157; 0.003; 0.054 (0.679)
** ** **PB**	141.4 ± 26.5	158.5 ± 54.1	179.5 ± 48.2	148.4 ± 37.9	130.4 ± 28.8	144.8 ± 34.1		
**CK (IU/L)**								
** ** **CON**	170 ± 101	203 ± 108	257 ± 130	468 ± 575	320 ± 383	218 ± 182	F = 13.364; F = 0.0001; F = 0.155 *p* < 0.001; *p* = 0.990; *p* = 0.978	0.386; 0.001; 0.007 (0.775)
** ** **PB**	179 ± 117	211 ± 123	273 ± 156	368 ± 253	235 ± 141	240 ± 202		
**LDH (IU/L) †**								
** ** **CON**	151 ± 24	170 ± 24	169 ± 21	159 ± 24	156 ± 26	157 ± 24	χ^2^ = 52.16; χ^2^ = 1.65; χ^2^ = 2.95 *p* < 0.001; *p* = 0.198; *p* = 0.707	(0.579)
** ** **PB**	164 ± 17	187 ± 20	188 ± 23	169 ± 27	162 ± 21	185 ± 59		
**PC (nmol/mg) †**								
** ** **CON**	0.34 ± 0.03	0.32 ± 0.04	0.33 ± 0.04	0.33 ± 0.03	0.33 ± 0.02	0.33 ± 0.04	χ^2^ = 18.6; χ^2^ = 14.5; χ^2^ = 15.8 # *p* = 0.002; *p* < 0.001; *p* = 0.007	(0.802)
** ** **PB**	0.23 ± 0.05	0.23 ± 0.06	0.27 ± 0.07	0.22 ± 0.06	0.22 ± 0.06	0.23 ± 0.07		
**Isoprostanes (pg/mg/creatinine) †**								
** ** **CON**	11.4 ± 3.42	12.7 ± 4.6	15.0 ± 8.9	10.3 ± 4.0	19.3 ± 26.0	11.9 ± 3.2	χ^2^ = 8.04; χ^2^ = 0.008; χ^2^ = 2.37 *p* = 0.154; *p* = 0.925; *p* = 0.795	(0.298)
** PB**	12.5 ± 6.7	16.3 ± 12.7	13.4 ± 8.2	11.7 ± 4.2	16.3 ± 11.5	14.4 ± 11.4		

CK, creatine kinase; Hs-CRP, high sensitivity c-reactive protein; LDH, lactate dehydrogenase; IL-6, interleukin-6; MCP-1, monocyte chemoattractant protein-1; PC, protein carbonyls; ηp^2^ = partial eta squared, presented for linear mixed model (LMM) analysis only. Pre = pre-exercise; Post = post-exercise. # All *p* > 0.05 when analysed with pre-exercise as a covariate. R^2^ = conditional R^2^ for LMM and generalised linear mixed model (GLMM). † GLMM analysis performed as data not normally distributed. χ^2^ statistic presented for GLMM analysis. Data are means ± standard deviation.

## Data Availability

The original contributions presented in this study are included in the article/Supplementary Material. Further inquiries can be directed to the corresponding author.

## Supplementary Materials

The following supporting information can be downloaded at: https://www.mdpi.com/article/10.3390/nu18081199/s1, File S1: CONSORT 2025 checklist. Reference [57] is cited in the Supplementary Materials.
